# Risk Factors for Anastomotic Leakage Following Stoma Closure: A Retrospective Study in Tertiary Hospitals in Yemen

**DOI:** 10.7759/cureus.75407

**Published:** 2024-12-09

**Authors:** Mohammed Al-Shehari, Yasser A Obadiel, Matheel M Abdulwahab, Haitham M Jowah

**Affiliations:** 1 Department of Surgery, Faculty of Medicine and Health Science, Sana'a University, Sana'a, YEM; 2 Department of Surgery, Al-Thawra Modern General Hospital, Sana'a, YEM; 3 Department of Surgery, Republican Teaching Hospital Authority, Sana'a, YEM

**Keywords:** anastomotic leak, risk factors, stoma closure, surgical outcomes, yemen

## Abstract

Introduction

Anastomotic leakage (AL) following stoma closure is a significant complication that can lead to increased morbidity and mortality. Identifying risk factors associated with AL is essential for improving surgical outcomes, especially in resource-limited settings like Yemen.

Methods

We conducted this retrospective study at Al-Thawra Modern General Hospital and the Republican Teaching Hospital Authority in Sana’a, Yemen, between August 2020 and April 2024. The analysis included 50 patients aged 18-65 years who underwent stoma closure. We analyzed patient data, including demographics, comorbidities, surgical technique, and outcomes, to identify risk factors for AL.

Results

The incidence of AL was six (12%) out of 50 cases. Significant risk factors included smoking, with AL present in four (67%) smokers and two (33%) non-smokers (p = 0.045). Patients with diverticulitis were more likely to require a stoma in two (33%) cases, and perforated small bowel with peritonitis in one (17%) case, compared to trauma cases in two (7%) and colorectal cancer cases at one (11%) (p = 0.038). AL was most common in colorectal anastomosis, observed in four (67%) cases, compared to other sites in two (5%) cases (p = 0.001). The surgical technique impacted the incidence of AL, with hand-sewn anastomosis showing a higher rate in four (67%) cases compared to stapled anastomosis in two (33%) cases (p = 0.036). No significant associations were found for age, sex, American Society of Anesthesiologists (ASA) classification, or surgeon experience.

Conclusion

This study identifies key risk factors for AL following stoma closure in the context of hospitals in Yemen, emphasizing the need for targeted preoperative and intraoperative strategies, such as smoking cessation and careful surgical technique selection, to reduce the risk of AL. Future studies should focus on larger cohorts and the impact of enhanced perioperative care protocols in low-resource settings.

## Introduction

Stoma closure is a critical aspect of gastrointestinal surgery that aims to restore the natural continuity of the digestive tract after a temporary diversion. Although this procedure is routinely performed, it is not without significant risks, with the most severe being anastomotic leakage (AL). AL is defined as the leakage of luminal fluid at the anastomotic site and is associated with severe complications, including peritonitis, sepsis, and increased mortality. The incidence of AL varies widely, ranging from 3% to 11%, depending on patient demographics, surgical techniques, and healthcare settings [1,2].

Understanding the risk factors of AL is important for improving surgical outcomes and patient safety. Established risk factors include advanced age, comorbid conditions such as diabetes mellitus and hypertension, use of intraperitoneal drains, open surgery, surgical site infections, blood transfusions, involvement of the left colon, prior chemotherapy, and anticoagulant use [3-5]. Early diagnosis is pivotal for effective management, including clinical assessments, imaging techniques, and biomarkers like C-reactive protein [6]. Management strategies can vary, from conservative treatments to more invasive surgical interventions, depending on the severity of AL and the patient’s overall condition [7].

The majority of studies on AL have been conducted in high-resource settings, which may limit their applicability to low-resource environments, such as Yemen, where healthcare infrastructure and patient demographics differ significantly. In Yemen, limited access to advanced medical resources and variations in clinical practice can influence patient outcomes. Previous studies have identified risk factors such as male sex, obesity, prolonged operative time, preoperative steroid use, and radiochemotherapy as contributors to AL [8-10].

Recent research has suggested that inflammatory biomarkers and other indicators of systemic response after surgery could predict AL risk [11]. Identifying modifiable risk factors is crucial for developing targeted perioperative interventions aimed at reducing AL incidence and improving patient outcomes.

This study investigated the incidence and risk factors of AL following stoma closure at two major tertiary hospitals in Sana’a, Yemen. By identifying these factors, this study seeks to provide insights that can inform clinical practice in similar low-resource settings and enhance patient safety.

## Materials and methods

Study design and setting

This retrospective observational study was conducted in the general surgery departments of Al-Thawra Modern General Hospital and the Republican Teaching Hospital in Sana'a, Yemen, from August 2020 to April 2024. These hospitals are major tertiary care centers that handle a broad spectrum of surgical cases, including complex stoma closures. Given their capacity and the diversity of cases managed, these settings provided a robust patient cohort to examine postoperative outcomes associated with stoma closure, including the risk of anastomotic leakage.

Participants

This study included 50 consecutive patients who underwent stoma closure at either of the two participating hospitals during the study period. Eligible participants were adults aged 18-65 years who had undergone closure of any type of stoma, including colostomy, ileostomy, or other variants. Patients were excluded if they were younger than 18 years or older than 65 years, had incomplete medical records, or were immunocompromised, such as those receiving chemotherapy, on prolonged corticosteroids, or with autoimmune diseases requiring immunosuppressive therapy. These criteria were selected to ensure a homogeneous cohort and minimize potential confounding factors that could impact the study outcomes.

Data collection

Data for this study were retrospectively collected from the medical records of eligible patients using a standardized data collection form. A wide range of demographic, clinical, and procedural variables were gathered. These included basic demographic information such as age, sex, and smoking status, as well as detailed information about relevant comorbidities including diabetes mellitus, hypertension, and ischemic heart disease. Preoperative evaluations documented in the study included the American Society of Anesthesiologists (ASA) classification, preoperative albumin levels, and the time interval between stoma creation and closure. Intraoperative data focused on the type of stoma (such as end colostomy or loop ileostomy), the type of anastomosis (e.g., end-to-end or end-to-side), the site of anastomosis, the surgical technique (hand-sewn or stapled), and the level of surgeon experience (classified as either expert or resident). Postoperative data included outcomes such as the incidence of anastomotic leakage (AL), surgical site infections (SSI), length of hospital stay, and the management strategies employed for any identified complications.

Outcome measures

The primary outcome was the incidence of anastomotic leakage (AL) within 30 days after stoma closure, defined as any clinically significant leakage from the anastomosis site, confirmed by clinical signs, imaging, or reoperation. Secondary outcomes included early postoperative complications, such as surgical site infections (SSI), wound dehiscence, and other systemic complications like sepsis, occurring within the same 30-day period. SSI was defined by clinical signs of infection and wound dehiscence was identified by the separation of the surgical incision. These complications were monitored to assess their impact on recovery.

Statistical analysis

Data analysis was performed using SPSS Statistics version 22.0 (Armonk, NY: IBM Corp.). Categorical variables were summarized as frequencies and percentages, while continuous variables were expressed as means with standard deviations. To assess associations between categorical variables (such as smoking status, ASA classification, and surgical technique) and the incidence of anastomotic leakage, chi-square tests and Fisher's exact tests were employed. A significance level of p < 0.05 was set to determine statistically significant differences. This approach allowed for the identification of factors that were most strongly associated with AL and provided a clear understanding of potential risks for complications in this patient population.

Bias control

To mitigate the risk of selection bias, the study included all patients who met the eligibility criteria and underwent stoma closure during the study period. To address information bias, data were carefully extracted and cross-verified using standardized forms. This process ensured that the information collected was as accurate and complete as possible, which is particularly important in retrospective studies where incomplete or inconsistent records can undermine the validity of the findings.

Ethical considerations

Ethical approval for the study was granted by the Ethical Committee of Sana’a University, Sana’a, Yemen (#SU-2020-IRB-186). Informed consent was obtained from all patients included in the study. The research was conducted in full accordance with the ethical principles outlined in the Declaration of Helsinki, ensuring patient privacy and confidentiality throughout the data collection and analysis processes. These ethical safeguards were in place to protect the participants and to ensure that the study adhered to the highest standards of ethical research conduct.

## Results

**Figure 1:**
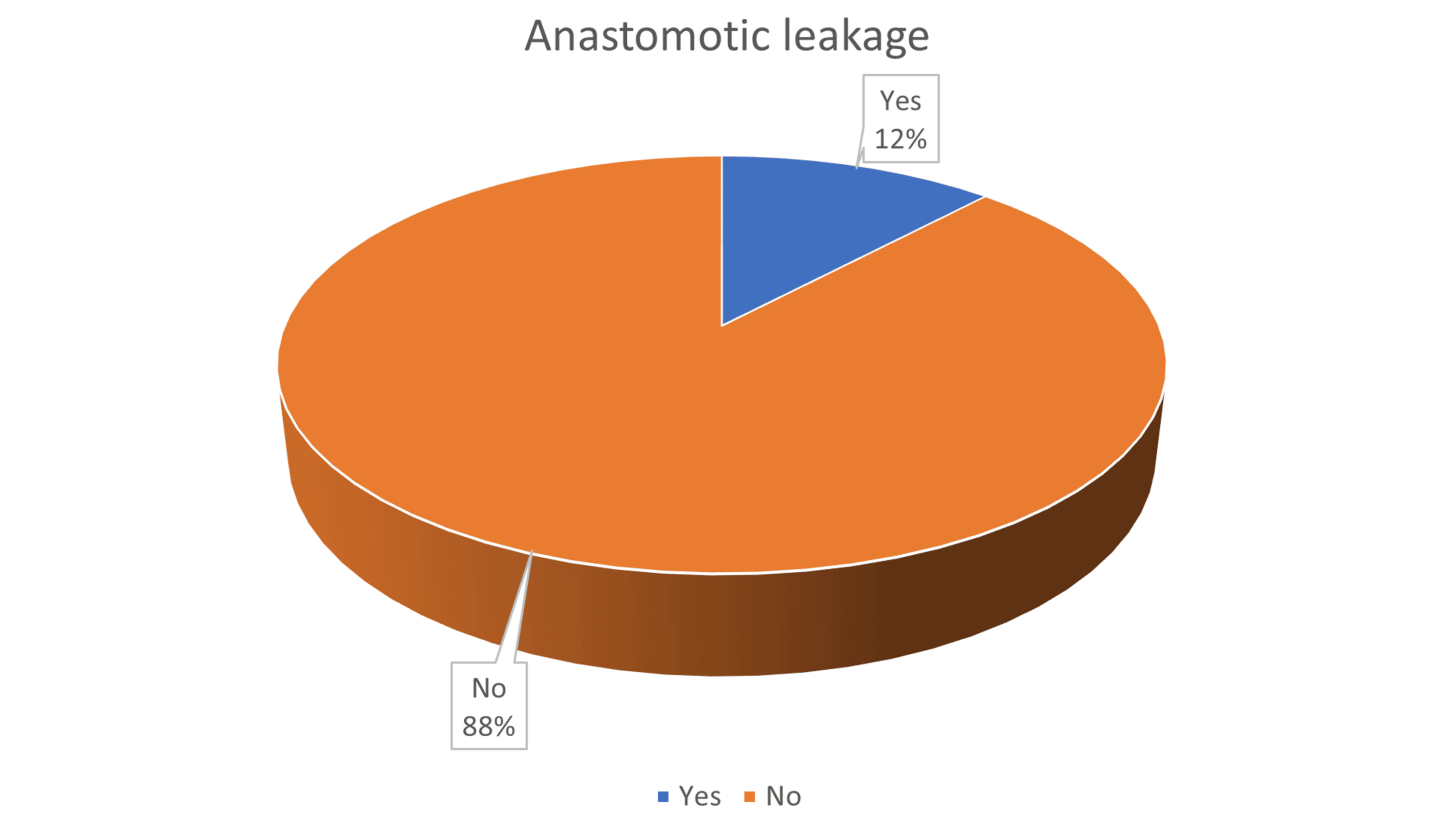
The incidence of anastomotic leakage among patients underwent stoma closure.

A total of 50 patients aged 18-65 years who underwent stoma closure were included in the study. The incidence of anastomotic leakage (AL) was observed in six (12%) out of 50 patients (Figure 1).

Demographic and clinical characteristics

Of the 50 patients, 47 (94%) were male, which reflects the higher incidence of trauma, the most common indication for stoma creation, seen predominantly in males. Trauma accounted for 28 (56%) cases, followed by colorectal cancer (18%), diverticulitis (12%), and perforated small bowel with peritonitis (2%). AL rates varied by age as follows: 9% in those aged 19-40 years, 17% in those aged 41-60 years, and 25% in patients aged 61-65 years. Smoking was present in 18 (36%) patients, with four (67%) AL cases occurring in smokers (p = 0.045).

Comorbidities included hypertension in seven (14%) patients (one {16.7%} with AL) and diabetes in four (8%) (two {33.3%} with AL). Ischemic heart disease was noted in one (2%) patient without AL. The majority of patients (70%) were classified as ASA 1, with AL rates higher in ASA 3 patients (50%). Preoperative albumin levels >3.5 mg/dL were seen in 60% of patients. Mechanical bowel preparation was performed in 62% of patients, with three (50%) AL cases in this group. The most common time between stoma creation and closure was one to three months (48%), with two AL cases during this period (Table 1).

**Table 1:** Demographic and clinical characteristics of patients. *P < 0.05 was considered statistically significant. P-values were calculated using chi-square tests for categorical variables to evaluate the association between each variable and AL. For variables with small sample sizes, Fisher's exact test was applied as needed. AL: anastomotic leakage; HTN: hypertension; DM: diabetes mellitus; IHD: ischemic heart disease; ASA: American Society of Anesthesiologists

Characteristic	AL present (n=6, %)	AL absent (n=44, %)	Total (n=50, %)	Chi-square value	p-Value
Sex					
Male	6 (12.8%)	41 (87.2%)	47 (94.0%)	0.44	0.509
Female	0 (0.0%)	3 (100.0%)	3 (6.0%)		
Age categories					
19-40 years	3 (8.8%)	31 (91.2%)	34 (68.0%)	1.21	0.545
41-60 years	2 (16.7%)	10 (83.3%)	12 (24.0%)		
61-65 years	1 (25.0%)	3 (75.0%)	4 (8.0%)		
Comorbidities					
Smoking	4 (66.7%)	14 (31.8%)	18 (36.0%)	2.78	0.045*
Hypertension (HTN)	1 (16.7%)	6 (13.6%)	7 (14.0%)	0.0	0.098
Diabetes mellitus (DM)	2 (33.3%)	2 (4.5%)	4 (8.0%)	2.68	0.260
Ischemic heart disease (IHD)	1 (16.7%)	0 (0.0%)	1 (2.0%)	1.40	0.107
ASA classification					
1	2 (5.7%)	33 (75.0%)	35 (70.0%)	4.68	0.098
2	1 (16.7%)	4 (9.1%)	5 (10.0%)		
3	3 (50.0%)	7 (15.9%)	10 (20.0%)		
Preoperative albumin levels					
2.5-3 mg/dL	2 (33.3%)	4 (66.7%)	6 (12.0%)	3.46	0.177
3-3.5 mg/dL	2 (14.3%)	12 (85.7%)	14 (28.0%)		
>3.5 mg/dL	2 (6.7%)	28 (93.3%)	30 (60.0%)		
Mechanical bowel preparation					
Yes	3 (50.0%)	28 (63.6%)	31 (62.0%)	0.04	0.315
No	3 (50.0%)	16 (36.4%)	19 (38.0%)		
Indication for stoma creation					
Trauma	2 (33.3%)	26 (59.1%)	28 (56.0%)	9.09	0.038*
Colorectal cancer	1 (16.7%)	8 (18.2%)	9 (18.0%)		
Diverticulitis	2 (33.3%)	4 (9.1%)	6 (12.0%)		
Perforated small bowel with peritonitis	1 (16.7%)	0 (0.0%)	1 (2.0%)		
Duration between stoma creation and closure					
<1 Month	1 (16.7%)	7 (15.9%)	8 (16.0%)	2.5	0.475
1-3 Months	2 (33.3%)	22 (50.0%)	24 (48.0%)		
3-6 Months	3 (50.0%)	10 (22.7%)	13 (26.0%)		
6 Months-1 year	0 (0.0%)	5 (11.4%)	5 (10.0%)		

Risk factors for anastomotic leakage

Smoking was significantly associated with anastomotic leakage (AL), with four (66.7%) of six AL cases occurring in smokers (p = 0.045). The indication for stoma creation also influenced AL, with the highest rates in patients with stomas for trauma (33.3%) and diverticulitis (33.3%) (p = 0.038). End colostomies had the highest AL incidence (83.3%), while loop ileostomies had no AL cases (p = 0.155). The site of the anastomosis was a strong factor, with four (66.7%) of six AL cases at the colorectal site (p = 0.001). Hand-sewn anastomoses accounted for four (66.7%) AL cases, while stapled anastomoses contributed two (33.3%) (p = 0.036). Other factors, including ASA classification (p = 0.098), duration between stoma creation and closure (p = 0.475), surgeon experience (p = 0.752), preoperative albumin levels (p = 0.177), type of anastomosis (p = 0.867), and blood transfusion (p = 0.065), showed no significant association with AL (Table 2).

**Table 2:** Associated risk factors with anastomotic leakage using chi-square test. *P < 0.05 was considered statistically significant. This table highlights only the significant risk factors for anastomotic leakage identified in the analysis. Counts and percentages are based on the total number of patients in each category. The chi-square values were calculated to confirm the statistical significance of each variable.

Variables	AL present (n=6, %)	AL absent (n=44, %)	Total (n=50, %)	Chi-square value	p-Value
Smoking status					
Smoker	4 (66.7%)	14 (31.8%)	18 (36.0%)	2.78	0.045*
Non-smoker	2 (33.3%)	30 (68.2%)	32 (64.0%)		
ASA classification					
ASA 1	2 (33.3%)	33 (75.0%)	35 (70%)	1.00	0.098
ASA 2	1 (16.7%)	4 (9.1%)	5 (10%)		
ASA 3	3 (50.0%)	7 (15.9%)	10 (20%)		
Duration between stoma creation and closure					
<1 Month	1 (16.7%)	7 (15.9%)	8 (16%)	2.5	0.475
1-3 Months	2 (33.3%)	22 (50.0%)	24 (48%)		
3-6 Months	3 (50.0%)	10 (22.7%)	13 (26%)		
6 Months-1 year	0 (0.0%)	5 (11.4%)	5 (10%)		
Type of stoma					
End colostomy	5 (83.3%)	16 (36.4%)	21 (42%)	5.28	0.155
End ileostomy	1 (16.7%)	11 (25.0%)	12 (24%)		
Loop colostomy	0 (0.0%)	7 (15.9%)	7 (14%)		
Loop ileostomy	0 (0.0%)	10 (22.7%)	10 (20%)		
Indication for stoma creation					
Trauma	2 (33.3%)	26 (59.1%)	28 (56%)	9.09	0.038*
Colorectal cancer	1 (16.7%)	8 (18.2%)	9 (18%)		
Intestinal obstruction	0 (0.0%)	2 (4.5%)	2 (4%)		
Diverticulitis	2 (33.3%)	4 (9.1%)	6 (12%)		
Perforated appendix	0 (0.0%)	3 (6.8%)	3 (6%)		
Midgut volvulus	0 (0.0%)	1 (2.3%)	1 (2%)		
Perforated small bowel with peritonitis	1 (16.7%)	0 (0.0%)	1 (2%)		
Preoperative albumin					
2.5-3 mg/dL	2 (33.3%)	4 (9.1%)	6 (12%)	3.46	0.177
3-3.5 mg/dL	2 (33.3%)	12 (27.3%)	14 (28%)		
>3.5 mg/dL	2 (33.3%)	28 (63.6%)	30 (60%)		
Surgeon experience					
Resident	3 (50.0%)	19 (43.2%)	22 (44%)	0.16	0.752
Expert	3 (50.0%)	25 (56.8%)	28 (56%)		
Site of anastomosis					
Ileoileal	1 (16.7%)	14 (31.8%)	15 (30%)	6.37	0.001*
Colocolic	0 (0.0%)	22 (50.0%)	22 (44%)		
Ileo-transverse	1 (16.7%)	5 (11.4%)	6 (12%)		
Colorectal	4 (66.7%)	3 (6.8%)	7 (14%)		
Type of anastomosis					
End to end	5 (83.3%)	38 (86.4%)	43 (86%)	0.16	0.867
End to side	1 (16.7%)	5 (11.4%)	6 (12%)		
Side to side	0 (0.0%)	1 (2.3%)	1 (2%)		
Surgical technique for stoma closure					
Hand-sewn anastomosis	4 (66.7%)	40 (90.9%)	44 (88%)	4.39	0.036*
Stapled anastomosis	2 (33.3%)	4 (9.1%)	6 (12%)		
Intraoperative blood loss and transfusion					
Yes (significant blood loss >500 mL)	5 (83.3%)	19 (43.2%)	24 (48%)	3.34	0.065
No (minimal blood loss ≤500 mL)	1 (16.7%)	25 (56.8%)	26 (52%)		

Postoperative complications and outcomes

Surgical site infections (SSI) occurred in eight out of 50 patients (16%), with a higher prevalence among those with anastomotic leakage (AL). The length of hospital stay varied, with 27 patients (54%) staying less than one week and two patients (4%) staying more than three weeks (Table 3). Prolonged admissions were primarily associated with postoperative complications, including AL and SSIs, which necessitated extended monitoring, additional interventions, and delayed recovery. Factors such as the complexity of stoma reversal, patient comorbidities, and perioperative management also contributed to extended stays in some cases.

**Table 3:** Postoperative complications and outcomes.

Complication/outcome	AL present (n=6, %)	AL absent (n=44, %)	Total (n=50, %)
Surgical site infection	4 (66.7%)	4 (9.1%)	8 (16.0%)
Length of hospital stay			
<1 Week	2 (33.3%)	25 (56.8%)	27 (54.0%)
1-2 Weeks	2 (33.3%)	15 (34.1%)	17 (34.0%)
2-3 Weeks	1 (16.7%)	3 (6.8%)	4 (8.0%)
>3 Weeks	1 (16.7%)	1 (2.3%)	2 (4.0%)

Management of anastomotic leakage

**Figure 2:**
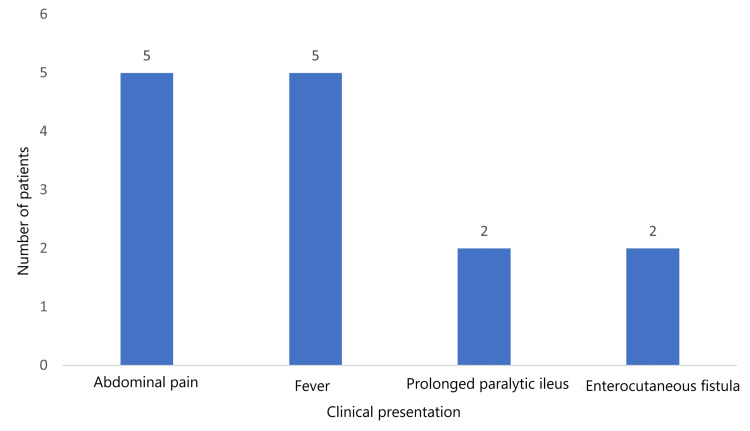
Frequency of clinical presentations in patients with anastomotic leakage poststoma closure.

Among the six cases of AL, the most common clinical presentations were abdominal pain and fever, each occurring in five (83.3%) cases. Two (33.3%) cases each showed prolonged paralytic ileus and enterocutaneous fistula, respectively (Figure 2).

**Figure 3:**
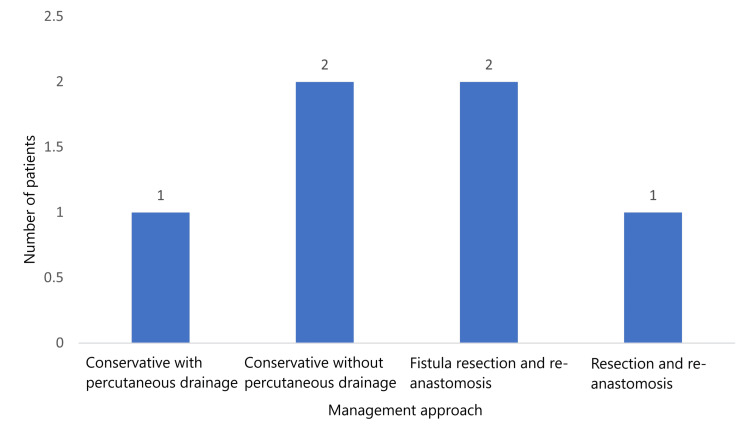
Frequency of management approaches for anastomotic leakage patients poststoma closure.

Management strategies included conservative treatment with antibiotics, either with percutaneous drainage in one (16.7%) case or without percutaneous drainage in two (33.3%) cases. Surgical interventions included fistula resection and re-anastomosis in two (33.3%) cases, and resection and re-anastomosis in one (16.7%) case (Figure 3).

## Discussion

The present study reports a 12% incidence of anastomotic leakage (AL) following stoma closure among patients treated at Al-Thawra Modern General Hospital and the Republican Teaching Hospital in Sana'a, Yemen. This rate aligns with the higher end of the reported range in the literature, which varies from 3% to 11% [2,3]. The relatively elevated incidence in our study highlights the need for heightened awareness and targeted preventive measures in this patient population.

A significant association between smoking and AL was identified in this study, consistent with existing evidence that links smoking with impaired wound healing and an increased risk of postoperative complications. Smoking-induced vasoconstriction and reduced tissue oxygenation can compromise the integrity of anastomoses, leading to higher leakage rates. Previous studies by Sørensen et al. and Baucom et al. have also documented smoking as a significant risk factor for AL after colorectal procedures [12,13]. These findings underscore the necessity of incorporating smoking cessation programs as part of preoperative patient optimization.

In the present study, we found that trauma, colorectal cancer, and diverticulitis were significant indications for stoma creation and were associated with the development of AL. These findings are consistent with previous reports indicating that underlying conditions affecting tissue quality and inflammation can predispose patients to AL [14]. Colorectal cancer and diverticulitis, in particular, are associated with compromised tissue integrity and increased inflammation, which can impair healing. The nature of the pathology, such as the extent of disease and the inflammatory status at the time of surgery, is crucial in determining the healing potential and susceptibility to AL. We suggest that future studies consider these pathologies in greater detail to elucidate their specific roles in AL.

Regarding stoma type, our study found that colorectal anastomoses had a higher risk of leakage compared to other sites, a finding that supports research by Park et al. and Telem et al., who highlighted that lower perfusion levels and the presence of fecal content in colorectal anastomoses contribute to an increased risk of leakage [5,11]. This underscores the importance of rigorous assessment of anastomotic perfusion and integrity during colorectal surgeries. Moreover, further clarification of the specific stoma type (e.g., loop ileostomy vs. end colostomy) and its relationship to AL rates would strengthen future studies.

The surgical technique employed also influenced the incidence of AL in our study. Hand-sewn anastomoses were associated with a higher incidence of AL compared to stapled techniques. Although hand-sewn closures are often preferred for their flexibility and control, they may be more technically demanding and susceptible to inconsistencies, potentially leading to higher leakage rates. This contrasts with the perception that stapled closures, while providing consistent and rapid results, may be associated with fewer complications in certain contexts. Factors contributing to higher AL rates in hand-sewn techniques could include operator variability and the complexity of achieving uniform tension along the suture line. Additionally, intraoperative techniques such as endoscopy and Doppler ultrasound remain valuable tools for assessing perfusion and anastomotic stability, as noted by Sánchez-Guillén et al. [15]. Future studies should continue to explore how surgical expertise, specific procedural nuances, and intraoperative tests impact outcomes.

The identification of significant risk factors such as smoking and surgical techniques highlights the need for targeted interventions. Smoking cessation programs and careful consideration of surgical methods could help reduce the risk of AL. Additionally, recognizing patients with high-risk indications for stoma creation, such as colorectal cancer or diverticulitis, can inform tailored perioperative management strategies. A more standardized approach to intraoperative monitoring and the consideration of nutritional status and preoperative optimization might further reduce the incidence of AL.

Future research should focus on the use of predictive biomarkers for assessing AL risk preoperatively. Biomarkers such as C-reactive protein (CRP) and procalcitonin have shown potential in predicting AL and could support more personalized patient management [16,17]. Innovative surgical techniques and materials that promote anastomotic healing, such as near-infrared fluorescence imaging for assessing tissue perfusion intraoperatively, also warrant further exploration [18]. Longitudinal studies are needed to evaluate the long-term effects of AL on patient outcomes, including survival and quality of life, given the association between AL and increased cancer recurrence [19]. Furthermore, the implementation and effectiveness of enhanced recovery after surgery (ERAS) protocols in reducing AL rates, particularly in resource-limited settings, should be assessed [20].

Limitations of the study

Despite the valuable insights provided by this study, certain limitations should be acknowledged. First, the retrospective design may have introduced selection and information biases, potentially impacting the accuracy of recorded data. Second, the relatively small sample size may limit the generalizability of the findings and reduce the statistical power for detecting associations with less common risk factors. Additionally, incomplete patient records, particularly with respect to variables such as body mass index (BMI) and detailed nutritional status, constrained the comprehensive analysis of all potential risk factors. These limitations highlight the need for larger, prospective studies to confirm the findings and explore additional risk factors for AL.

## Conclusions

This study identified smoking status, specific indications for stoma creation, site of anastomosis, and surgical technique as significant risk factors for AL following stoma closure. These findings highlight the importance of targeted perioperative strategies and smoking cessation programs to reduce AL risk. Addressing these risk factors could improve surgical outcomes, particularly in resource-limited settings such as Yemen.

## References

[1] Tsai YY, Chen WT (2019). Management of anastomotic leakage after rectal surgery: a review article. J Gastrointest Oncol.

[2] Pokorny H, Herkner H, Jakesz R, Herbst F (2005). Mortality and complications after stoma closure. Arch Surg.

[3] Kamarajah SK, Lin A, Tharmaraja T (2020). Risk factors and outcomes associated with anastomotic leaks following esophagectomy: a systematic review and meta-analysis. Dis Esophagus.

[4] Luján JJ, Németh ZH, Barratt-Stopper PA, Bustami R, Koshenkov VP, Rolandelli RH (2011). Factors influencing the outcome of intestinal anastomosis. Am Surg.

[5] Park JS, Choi GS, Kim SH (2013). Multicenter analysis of risk factors for anastomotic leakage after laparoscopic rectal cancer excision: the Korean laparoscopic colorectal surgery study group. Ann Surg.

[6] An V, Chandra R, Lawrence M (2018). Anastomotic failure in colorectal surgery: where are we at?. Indian J Surg.

[7] Vasiliu EC, Zarnescu NO, Costea R, Neagu S (2015). Review of risk factors for anastomotic leakage in colorectal surgery. Chirurgia (Bucur).

[8] Kang CY, Halabi WJ, Chaudhry OO (2013). Risk factors for anastomotic leakage after anterior resection for rectal cancer. JAMA Surg.

[9] Herrle F, Sandra-Petrescu F, Weiss C, Post S, Runkel N, Kienle P (2016). Quality of life and timing of stoma closure in patients with rectal cancer undergoing low anterior resection with diverting stoma: a multicenter longitudinal observational study. Dis Colon Rectum.

[10] Tsalikidis C, Mitsala A, Mentonis VI, Romanidis K, Pappas-Gogos G, Tsaroucha AK, Pitiakoudis M (2023). Predictive factors for anastomotic leakage following colorectal cancer surgery: where are we and where are we going?. Curr Oncol.

[11] Telem DA, Chin EH, Nguyen SQ, Divino CM (2010). Risk factors for anastomotic leak following colorectal surgery: a case-control study. Arch Surg.

[12] Sørensen LT, Jørgensen T, Kirkeby LT, Skovdal J, Vennits B, Wille-Jørgensen P (1999). Smoking and alcohol abuse are major risk factors for anastomotic leakage in colorectal surgery. Br J Surg.

[13] Baucom RB, Poulose BK, Herline AJ, Muldoon RL, Cone MM, Geiger TM (2015). Smoking as dominant risk factor for anastomotic leak after left colon resection. Am J Surg.

[14] Pigazzi A, Valero G, Weber R (2024). Assessing techniques to prevent anastomotic leak - advances in gastroenterology and GI surgery. https://www.nyp.org/advances/article/gastroenterology/assessing-techniques-to-prevent-anastomotic-leak.

[15] Sánchez-Guillén L, Frasson M, García-Granero Á, Pellino G, Flor-Lorente B, Álvarez-Sarrado E, García-Granero E (2019). Risk factors for leak, complications and mortality after ileocolic anastomosis: comparison of two anastomotic techniques. Ann R Coll Surg Engl.

[16] Su'a BU, Mikaere HL, Rahiri JL, Bissett IB, Hill AG (2017). Systematic review of the role of biomarkers in diagnosing anastomotic leakage following colorectal surgery. Br J Surg.

[17] Gray M, Marland JR, Murray AF, Argyle DJ, Potter MA (2021). Predictive and diagnostic biomarkers of anastomotic leakage: a precision medicine approach for colorectal cancer patients. J Pers Med.

[18] van den Bos J, Al-Taher M, Schols RM, van Kuijk S, Bouvy ND, Stassen LP (2018). Near-infrared fluorescence imaging for real-time intraoperative guidance in anastomotic colorectal surgery: a systematic review of literature. J Laparoendosc Adv Surg Tech A.

[19] Mohamed KB, Hansen CH, Krarup PM, Fransgård T, Madsen MT, Gögenur I (2020). The impact of anastomotic leakage on recurrence and long-term survival in patients with colonic cancer: a systematic review and meta-analysis. Eur J Surg Oncol.

[20] Bakker N, Deelder JD, Richir MC, Cakir H, Doodeman HJ, Schreurs WH, Houdijk AP (2016). Risk of anastomotic leakage with nonsteroidal anti-inflammatory drugs within an enhanced recovery program. J Gastrointest Surg.

